# Disability and sexual and reproductive health service utilisation in Uganda: an intersectional analysis of demographic and health surveys between 2006 and 2016

**DOI:** 10.1186/s12889-022-12708-w

**Published:** 2022-03-04

**Authors:** Muriel Mac-Seing, Christina Zarowsky, Mengru Yuan, Kate Zinszer

**Affiliations:** 1 Department of Social and Preventive Medicine, School of Public Health, Université de Montréal, Montreal, Canada; 2 Centre de recherche en santé publique, Université de Montréal et CIUSS du Centre-Sud-de-l’Île-de-Montréal, Montreal, Canada; 3 School of Public Health, University of Western Cape, Bellville, South Africa; 4 Department of Epidemiology, Biostatistics, and Occupational Health, McGill University, Montreal, Canada

**Keywords:** Determinants of health, Disability, Demographic and health surveys, Sexual and reproductive health service utilisation, Intersectionality, Health equity, Uganda

## Abstract

**Background:**

The United Nations through universal health coverage, including sexual and reproductive health (SRH), pledges to include all people, leaving no one behind. However, people with disabilities continue to experience multiple barriers in accessing SRH services. Studies analysing the impacts of disability in conjunction with other social identities and health determinants reveal a complex pattern in SRH service use. Framed within a larger mixed methods study conducted in Uganda, we examined how disability, among other key social determinants of health (SDH), was associated with the use of SRH services.

**Methods:**

We analysed data from repeated cross-sectional national surveys, the Uganda Demographic and Health Surveys (DHS) of 2006, 2011, and 2016. The three outcomes of interest were antenatal care visits, HIV testing, and modern contraception use. Our main exposure of interest was the type of disability, classified according to six functional dimensions: seeing, hearing, walking/climbing steps, remembering/concentrating, communicating, and self-care. We performed descriptive and multivariable logistic regression analyses, which controlled for covariates such as survey year, sex, age, place of residence, education, and wealth index. Interaction terms between disability and other factors such as sex, education, and wealth index were explored. Regression analyses were informed by an intersectionality framework to highlight social and health disparities within groups.

**Results:**

From 2006 to 2016, 15.5-18.5% of study participants lived with some form of disability. Over the same period, the overall prevalence of at least four antenatal care visits increased from 48.3 to 61.0%, while overall HIV testing prevalence rose from 30.8 to 92.4% and the overall prevalence of modern contraception use increased from 18.6 to 34.2%. The DHS year, highest education level attained, and wealth index were the most consistent determinants of SRH service utilisation. People with different types of disabilities did not have the same SRH use patterns. Interactions between disability type and wealth index were associated with neither HIV testing nor the use of modern contraception. Women who were wealthy with hearing difficulty (Odds Ratio (OR) = 0.15, 95%CI 0.03 – 0.87) or with communication difficulty (OR = 0.17, 95%CI 0.03 – 0.82) had lower odds of having had optimal antenatal care visits compared to women without disabilities who were poorer.

**Conclusion:**

This study provided evidence that SRH service use prevalence increased over time in Uganda and highlights the importance of studying SRH and the different disability types when examining SDH. The SDH are pivotal to the attainment of universal health coverage, including SRH services, for all people irrespective of their social identities.

## Introduction

Heads of State at the United Nations (UN)‘s 2019 High-Level Meeting reaffirmed their commitment to Sustainable Development Goal (SDG) 3.8 on universal health coverage (UHC), including equitable access for all to sexual and reproductive health (SRH) services and information [1]. Although UHC pledges to “leave no one behind”, disability is not a focus of the UHC despite being an important dimension of inclusion for the SDGs [2]. Globally, approximately 15% of the world’s population live with some form of disability, with 80% of these individuals residing in low- and middle-income countries (LMIC) [3]. According to the United Nations Convention on the Rights of Persons with Disabilities, people with disabilities include “those who have long-term physical, mental, intellectual or sensory impairments which in interaction with various barriers may hinder their full participation and effective participation in society on an equal basis with others” [4]. Several studies conducted in LMICs report that women, men, and youth with disabilities continue to encounter numerous obstacles, such as physical inaccessibility, disability-insensitive healthcare services, and negative attitudes of health staff and community members, in accessing SRH services such as antenatal care [5–8], contraception [7, 9, 10], HIV testing [5, 11, 12], and SRH information [7–10]. Although there is limited literature documenting the situation of people with disabilities beyond the focus on “medical and rehabilitative provision for conflict-related direct physical impairment” [13], it is suggested that in conflict or post-conflict settings, women with disabilities can face an additional risk of violence from community members [14]. A recent systematic review conducted in 11 sub-Saharan countries reported that people with disabilities faced multiple barriers to accessing SRH services, spanning the individual (e.g. gender) to the community (e.g. lack of community support), healthcare system (e.g. low capacity of staff), and economic levels (e.g. cost of service) [15].

Due to the multiple challenges experienced, people with disabilities have been reported to have poorer health outcomes [3, 16]. The literature identifies numerous determinants that influence access to SRH services. They include women’s age [17], education level [17, 18] and marital status [17], religion [19] as well as the location of residence [17, 20], and level of household wealth [17, 18, 21, 22]. However, studies analysing the impacts of disability in conjunction with other key social identities [6, 23, 24] report different patterns of associations with selected SRH utilisation outcomes. A cross-sectional study conducted in Sierra Leone found no significant difference between women with disabilities and those without disabilities when they sought maternal healthcare services, such as contraception use [23]. Using data from the Demographic and Health Survey (DHS), another study examined antenatal care among women with and without disabilities in Pakistan [6]. It reported that the overall measure of disability showed no association with antenatal care, while women with any severe disability had higher odds of receiving advice on exclusive breastfeeding than non-disabled women. In Cameroon, a study conducted among people with and without disabilities reported that although people with disabilities were at higher risk of poorer access to SRH services, disparities varied based on gender and disability [24]. The results of this study demonstrated that both women and men with disabilities had lower use of family planning and HIV testing that were not associated with access to SRH services, but were attributed to other factors associated with determinants related to respondents’ childhood, such as poorer access to education and work opportunities [24].

The Northern region of Uganda was most affected by two decades (1987-2006) of armed conflict in Uganda, with persisting weakened socioeconomic and health systems [25]. In 2006, when the conflict ended, the Disability Act was also adopted in Uganda to protect and promote the rights of people with disabilities [26]. Framed within this background of conflict and discrepancies in evidence related to people with disabilities’ access to SRH services, we investigated how disability, among other social health-related factors, is associated with SRH service utilisation. An exploratory qualitative study embedded in a larger mixed methods study found that adult women and men with disabilities living in Northern Uganda faced multiple challenges when using SRH services such as maternal care, contraception use, and HIV testing [11]. Major themes from the study included the complex intersections of disability with gender, HIV, and experience of violence [11]. The next step was then to investigate whether the perceived lack of access to and use of SRH services by people with disabilities in Northern Uganda was also observed nationally. The main objective of this present study was to examine how disability was associated with selected SRH service utilisation in Uganda between 2006 and 2016. In addition, informed by our qualitative study, we looked at the interactions between disability type and sex, education, wealth, and violence. Both studies (the previous qualitative study [11] and this current research) used an intersectionality-informed analysis to explore the co-existence of multiple social identities including disability [27] in relation to an important global public health issues: SRH [11, 28–30]. The intersectional approach highlights social and health inequities experienced by vulnerable populations such as marginalised pregnant women, youth, people of colour, and people with disabilities [31].

## Methods

### Study design and population

DHS datasets were made publicly available after the Uganda Bureau of Statistics processed and cleaned the data [32–34]. We analysed secondary data from three waves of Ugandan cross-sectional Demographic and Health Surveys (2006, 2011, and 2016). These national DHS were representative surveys at the regional level, using a stratified two-stage sample design [32–34]. Administratively, in the 2006 DHS, there were nine regions: Kampala (the capital), North, Central 1, Central 2, East Central, Eastern, West Nile, Western, and Southwest. In the 2011 DHS, the Northern region was split into two, adding a tenth region, Karamoja. In the 2016 DHS, these 10 regions were further divided into 15 regions, while keeping the outer geographical boundaries of 2006. Women participants’ data were obtained from the DHS Individual Recode Files, men’s data were obtained from the Men’s Files, and disability-related data were found in the DHS Household Files. All observations were combined in one dataset where we created the variables for sex and DHS year (2006, 2011, and 2016).

Given the importance of the experience of violence expressed by people with disabilities in our qualitative study [11], this study included participants who answered the Domestic Violence Module within each DHS which focused on adult women and men, aged 18 to 49 years old. In the 2006 DHS, one female participant in every three households responded to the Module questions, while one male respondent was selected among the remaining two households [32]. In the 2011 DHS, one woman per household was selected among the two-third of the households, while one man per household was selected in the remaining one-third of the households [33]. In the 2016 DHS, all households were invited to participate in the Module: one woman per household was randomly selected in two-thirds of the households, and in the remaining one-third of the households, a man per household responded to the questions [34]. In all three DHS, ever-married people were eligible for the Domestic Violence Module. Once privacy was ensured during the interviews, respondents answered questions related to emotional, physical, and sexual violence, as part of the Module [32–34]. The participation rate was 96.2, 99.3, and 99% among eligible women and 98.2, 98.8, and 99% among eligible men, in 2006, 2011, and 2016, respectively [32–34]. The main reason reported for the non-participation of eligible people was the lack of privacy to complete the Domestic Violence Module [32–34].

**Fig. 1:**
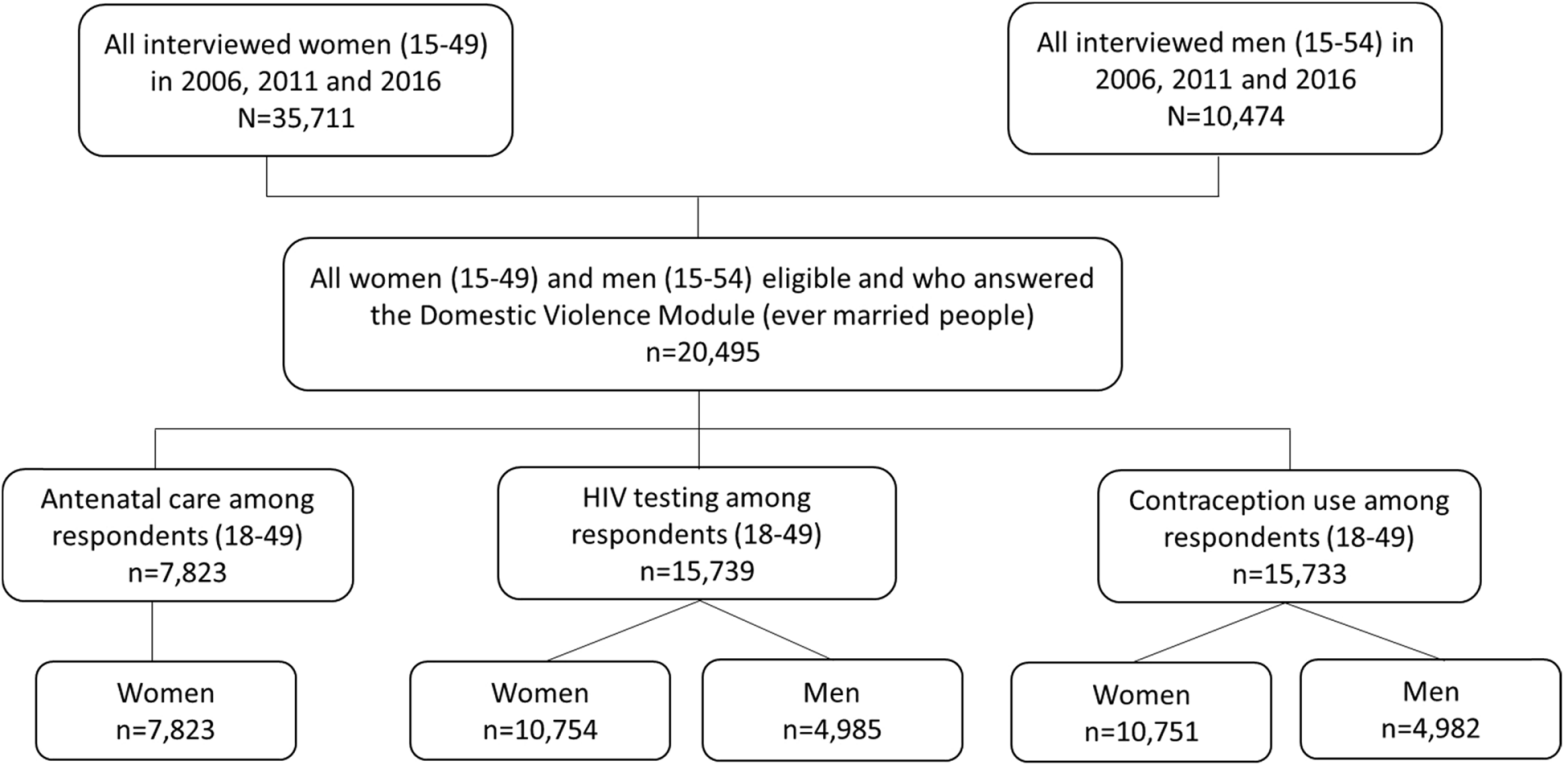
Study population

The study population included ever-married people aged 18-49 years old for a total of 7823 women for antenatal care visits, 10,754 women and 4985 men for HIV testing, and 10,751 women and 4982 men for contraception use over the three waves of data collection (Fig. 1). Although DHS data for men included those aged up to 54 years old, we included those aged 18-49 years old to be at par with the age brackets of women participants.

### Variables

The three outcomes included: 1) antenatal care visits for the last pregnancy, 2) HIV testing during the past year, and 3) use of current contraception type. The total number of antenatal care visits during their last pregnancy was recategorized as a binary variable based on the World Health Organization’s recommendation of at least four antenatal care visits for a positive pregnancy [35]: 0 for “0-3 antenatal care visits”, and 1 for “4 or more antenatal visits”. The HIV testing variable, which asked whether respondents have ever been tested for HIV during the past year of the survey, kept its binary form (0 = no, 1 = yes). For the current use of contraception method variable, participants were asked which method they were currently using at the time of the survey. Modern types of contraception (such as pills, injectables, male/female condoms and sterilisation, intrauterine devices, hormonal implants, and emergency contraception) were grouped together versus other methods (No use/use of traditional or folkloric contraception).

The main exposure variable of interest was the disability status, captured in the DHS as a ‘difficulty’ and following the Washington Group Short Set of Disability (WG) Questions [36]. The WG disability questions examined six functional dimensions: 1) seeing, 2) hearing, 3) walking or climbing steps, 4) remembering or concentrating, 5) self-care, and 6) communication, and according to four main levels of difficulty for each functional dimension: “No difficulty”, “Some difficulty”, “A lot of difficulty”, and “Cannot do it at all”. Disability type was recoded in a binary variable: 0 as “No difficulty”, and 1 as “At least some difficulty and above” which also included people who were reported to have severe difficulties in any of the functional dimensions.

Sensitivity analyses were conducted for antenatal care visits and disability, to examine if and how their categorization influenced the estimated effect measures. Other variables of interest included sex [37], age [17, 37], marital status [17], place of residence [20, 17], education level [17, 18], wealth index [20, 17, 21], religion [19], region (all regions in Uganda), and year of DHS (given the 10-year period we are studying). In addition, the experience of emotional, physical, and sexual violence was included as women and men with disabilities reported being at risk of and/or having experienced different forms of violence [11].

### Statistical analysis

The Uganda Bureau of Statistics processed and cleaned the DHS data before making them available publicly [32–34]. Data management, descriptive analyses, and multiple variable logistic regressions were conducted in R software (version 3.6.3) [38] and QGIS software (version 3.14) was used to produce bivariate choropleth maps [39]. Less than 1% of responses for outcomes were missing (respondents did not answer or did not know the answer) and were excluded from analyses. Descriptive analyses examined outcomes and exposure variables of interest at each time point. Bivariate choropleth maps were generated to examine how the overall disability status and outcomes of interest evolved by region between 2006 and 2016. To ensure comparability between the three different survey waves, we used the 2006 boundaries. Multivariable logistic regressions were created for each outcome of interest whereas a regional variable was created as well as survey year. Logistic regressions were adjusted for the two-stage sampling design used in the DHS using the ‘survey’ package in R. Variables with a Variance Inflation Factor (VIF) higher than 10, indicating the presence of multicollinearity, were excluded from analyses [40]. Given the intersectional approach adopted in our larger mixed methods study, key interaction terms (‘intersections’) informed by the qualitative findings were explored, emphasising the ‘multiplicative’ nature of people’s identities [41]. Specifically, we looked at interaction terms between disability type and each of the following: sex, education, wealth index, and experience of emotional, physical, and sexual violence. The selection of final models was based on the Akaike Information Criterion (AIC) and the residuals were examined for model fit [42]. In the three outcome models, we present final outputs and interaction terms of interest.

## Results

Table 1 summarises the socioeconomic characteristics of the study populations for selected SRH service use for the period between 2006 and 2016 in Uganda. The majority of respondents were women, ranging from 68.3% for HIV testing and use of modern contraception to 100% of respondents for antenatal care visits since direct maternal care only targeted women. Among the ever-married adult respondents of reproductive age (18-49 years old), approximately 11% were separated/divorced/widowed. Across SRH service use, 15.5-18.5% lived with some form of disability in at least one of the functional dimensions. Regarding difficulty type, 7.1-8.6% of respondents were reported having at least some difficulty in walking or climbing steps, and 7.6-8.6% had at least some difficulty in remembering or concentrating. People were also reported to have had at least some difficulty in seeing (2.7-4.0%), in hearing (1.2-1.4%), and in self-care (0.5-1.3%). Approximately 80% people lived in rural areas, had primary education (60.1-61.2%), and were of Anglican, Catholic, or Muslim faith. Approximately two-thirds of respondents were situated in the three lowest wealth quintiles. Approximately 40% of respondents experienced emotional violence, 35.8-42.8% faced physical violence, and 19.4-25.1% reported sexual violence.

**Table 1:** Characteristics of population by SRH service in Uganda (2006-2016)

	Antenatal care visits (*N* = 7823) Percentage	HIV testing and use of modern contraception type (*N* = 15,739^a^) Percentage
**Sex**		
Women	100	68.3
**Age in years**		
18-19	5.4	3.9
20-24	26.2	18.4
25-29	26.5	21.4
30-34	20.8	20.4
35-39	13.1	15.7
40 and >	8.0	20.1
**Marital status**		
Married/in union	89.3	87.1
Separated / divorced / widowed	10.7	12.9
**Disability**		
Overall	15.5	18.5
Difficulty seeing	2.7	4.0
Difficulty hearing	1.2	1.4
Difficulty walking / climbing steps	7.1	8.6
Difficulty remembering / concentrating	7.6	8.6
Difficulty with self-care	0.8	1.1
Difficulty communicating	0.9	1.2
**Highest education level attained**		
No education	15.9	13.6
Primary	61.2	60.1
Secondary	18.2	19.3
Higher	4.7	7.0
**Wealth index**		
Quintile 1 (poorest)	26.1	23.3
Quintile 2 (poorer)	22.1	21.1
Quintile 3 (middle)	18.2	18.4
Quintile 4 (richer)	16.8	18.0
Quintile 5 (richest)	16.7	19.2
**Religion**		
Anglican	35.5	36.5
Catholic	37.3	38.2
Muslim	12.7	11.9
Seven Day Adventist / Pentecostal / Born Again / Evangelical	12.5	11.5
Other	2.0	1.9
**Place of residence**		
Rural	81.7	79.9
**Region**		
Kampala	5.4	6.2
North	19.0	18.0
Central 1	8.4	8.8
Central 2	8.5	8.9
East Central	9.4	9.3
Eastern	16.5	16.0
West Nile	8.3	7.9
Western	12.0	12.2
Southeast	12.5	12.5
**Experience of violence**		
Emotional	42.1	40.8
Physical	42.8	35.8
Sexual	25.1	19.4

^a^ For the use of modern contraception type, there are six people less, *N* = 15,733

**Fig. 2:**
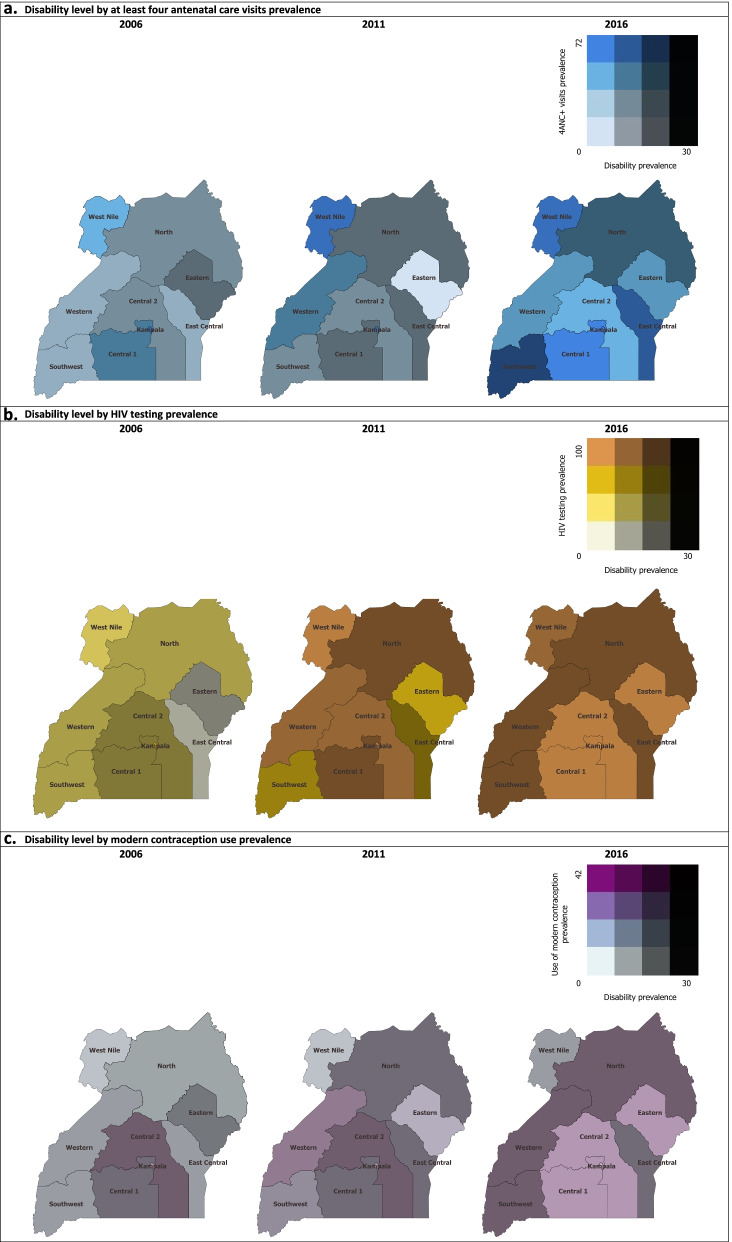
Disability level by SRH service use prevalence in Uganda between 2006 to 2016*. **a** Disability level by at least four antenatal care visits prevalence. **b** Disability level by HIV testing prevalence. **c** Disability level by modern contraception use prevalence. *For comparability, the 2006 boundaries of Uganda were used for the 2011 and 2016 maps

Regarding the three selected SRH services used over the years, between 2006 to 2016, the overall prevalence of at least four antenatal care visits increased from 48.3 to 61.0%, while overall HIV testing prevalence rose from 30.8 to 92.4% and the overall prevalence of modern contraception use increased from 18.6 to 34.2%. As per region and DHS year, the disability prevalence ranged from 7.9-29.6% (Fig. 2). Across the regions, the prevalence of at least four antenatal care visits increased most from 2011 to 2016 (Fig. 2a). HIV testing in 2006 was low with the exception in Kampala, where more than 60% of respondents reported having been tested for HIV (Fig. 2b). From 2011 to 2016, HIV testing increased to at least 80% in most of the regions. Regarding the use of modern contraception (Fig. 2c), slight changes were observed from 2006 to 2016 throughout the country.

### Determinants of sexual and reproductive health service utilisation

In Table 2, the disability type was not associated with the SRH service use, except for people with difficulty in communicating who had lower odds of having used modern contraception compared to people without disabilities (OR = 0.51, 95%CI 0.29 – 0.90). Women had higher odds of being tested for HIV (OR = 2.76, 95%CI 2.38 – 3.21), while sex was not associated with the use of modern contraception. People who were separated, divorced, or widowed had lower odds of having had the optimal number of antenatal care visits (OR = 0.76, 95%CI 0.63 – 0.90) and to have used modern contraception (OR = 0.76, 95%CI 0.67 – 0.86) relative to married/in union participants. Violence of any type was not associated with either the use of antenatal care or HIV testing. However, participants who experienced emotional (OR = 1.22, 95%CI 1.11 – 1.34) and physical violence (OR = 1.15, 95%CI 1.04 – 1.27) were more likely to have used modern contraception.

**Table 2:** Multiple logistic regression models on sexual and reproductive health use

	Model I^**a**^: At least four antenatal care visits		Model II^**b**^: HIV testing		Model III^**c**^: Modern contraception type use	
	OR	95% CI	OR	95% CI	OR	95% CI
**Disability (Ref**^**d**^**: No difficulty)**						
Difficulty seeing	1.09	0.76 – 1.57	1.17	0.82 – 1.66	0.98	0.70 – 1.39
Difficulty hearing	0.60	0.19 – 1.89	1.64	0.89 – 3.03	1.19	0.66 – 2.17
Difficulty walking / climbing steps	1.22	0.99 – 1.49	0.90	0.63 – 1.29	1.29	0.98 – 1.71
Difficulty remembering / concentrating	0.85	0.70 – 1.05	0.94	0.68 – 1.30	1.05	0.82 – 1.34
Difficulty in self-care	1.32	0.75 – 2.32	0.45	0.15 – 1.37	1.37	0.67 – 2.80
Difficulty in communicating	1.54	0.34 – 6.90	0.62	0.20 -1.96	0.51*^e^	0.29 – 0.90
**Year (Ref: 2006)**						
2011	1.03	0.85 – 1.25	8.78***	7.37 – 10.46	1.41***	1.21 – 1.65
2016	1.62***	1.38– 1.89	29.31***	24.93 – 34.45	2.29***	1.99 – 2.63
**Sex (Ref: Man for Models II and III)**						
Woman	–	–	2.76***	2.38 – 3.21	0.93	0.84 – 1.04
**Marital status (Ref: Married / in union)**						
Separated / divorced / widowed	0.76**	0.63 – 0.90	0.88	0.74 – 10.4	0.76***	0.67 – 0.86
**Religion (Ref: Anglican)**						
Catholic	–	–	–	–	0.92	0.84 – 1.02
Muslim	–	–	–	–	0.81**	0.70 – 0.94
Seven Day Adventist / Pentecostal / Born Again / Evangelical	–	–	–	–	0.76***	0.66 – 0.86
Other	–	–	–	–	0.63**	0.45 – 0.88
**Highest education (Ref: No education)**						
Primary	1.10	0.93 – 1.30	1.82***	1.53 – 2.15	1.88***	1.60 – 2.21
Secondary and higher	1.43***	1.18 – 1.87	3.46***	2.75 – 4.34	2.32***	1.92 – 2.81
**Wealth index (Ref: Quintile 1 Poorest)**						
Quintile 2 (poorer)	1.20*	1.01 – 1.42	1.14	0.96 – 1.35	1.41***	1.22 – 1.61
Quintile 3 (middle)	1.22	1.00 – 1.48	1.39***	1.15 – 1.69	1.58***	1.37 – 1.84
Quintile 4 (richer)	1.48***	1.22 – 1.83	1.66***	1.33 – 2.06	1.99***	1.70 – 2.33
Quintile 5 (richest)	1.81***	1.41 – 2.33	2.80***	2.10 – 3.73	2.09***	1.73 – 2.52
**Region (Ref: Urban)**						
Rural	1.02	0.84 – 1.22	0.95	0.71 – 1.21	0.86*	0.74 – 0.996
**Region (Ref: Kampala)**						
North	1.13	0.82 – 1.57	1.50	0.99 – 2.30	0.95	0.74 – 1.22
Central 1	0.88	0.63 – 1.24	0.76	0.50 – 1.16	0.83	0.65 – 1.07
Central 2	0.75	0.54 – 1.03	0.85	0.57 – 1.28	1.15	0.91 – 1.45
East Central	1.02	0.73 – 1.43	0.62*	0.41– 0.93	0.75*	0.57 – 0.97
Eastern	0.79	0.57 – 1.09	0.75	0.50 – 1.14	0.96	0.76 – 1.20
West Nile	1.52*	1.05 – 2.21	1.36	0.88 -2.10	0.50***	0.38 – 0.67
Western	0.94	0.67 – 1.31	0.86	0.58 – 1.28	0.89	0.70 – 1.13
Southeast	1.04	0.75 – 1.43	0.79	0.53 – 1.18	0.80	0.63 – 1.02
**Experienced violence (Ref: No)**						
Emotional violence	1.11	0.98 – 1.25	1.06	0.93 – 1.21	1.22***	1.11 – 1.34
Physical violence	0.90	0.78 – 1.02	0.92	0.80 – 1.06	1.15**	1.04 – 1.27
Sexual violence	0.93	0.81 – 1.06	0.91	0.79 – 1.05	–	
Disability type*Sex (Ref: Man and without any type of difficulty)						
Difficulty seeing*Sex	–	–	0.56*	0.35 – 0.90	1.33	0.85 – 2.09
Difficulty in self-care*Sex	–	–	3.58*	1.23 – 10.38	0.41	0.17 – 1.01
Disability type*Education (Ref: Without disability and no education)						
Difficulty hearing*Primary education	2.91	0.80 – 10.53	–	–	–	–
Difficulty hearing*Secondary education and higher	10.84*	1.67 – 70.54	–	–	–	–
Disability type*Wealth index (Ref: Without any type of difficulty and poorer)						
Difficulty hearing*Poor	0.37	0.10 – 1.37	–	–	–	–
Difficulty hearing*Middle	1.01	0.24 – 4.16	–	–	–	–
Difficulty hearing*Rich	0.15*	0.03 – 0.87	–	–	–	–
Difficulty hearing*Richer	0.16*	0.03 – 0.89	–	–	–	–
Difficulty in communicating*Poor	0.23	0.05 – 1.15	–	–	–	–
Difficulty in communicating*Middle	0.48	0.07 – 3.42	–	–	–	–
Difficulty in communicating*Rich	0.17*	0.03 – 0.82	–	–	–	–
Difficulty in communicating*Richer	0.90	0.07 – 12.32	–	–	–	–

^a^ Adjusted for disability type, year, marital status, residence, region, education, wealth index, age, and violence

^b^ Adjusted for disability type, year, sex, marital status, residence, region, education, wealth index, age, and violence

^c^ Adjusted for disability type, year, sex, marital status, religion, residence, region, education, wealth index, age, and violence

^d^ Reference group

^e^ *p < 0.05, **p < 0.01, ***p < 0.001 in two-tailed tests of significance

There were three covariates that showed a consistent association with the SRH service use: the DHS year, education level, and wealth index. Compared to 2006, the year 2016 showed higher odds of having had at least four antenatal care visits (OR = 1.62, 95%CI 1.38 – 1.89), of being tested for HIV (OR = 29.31, 95%CI 24.93 – 34.35), and having used modern contraception (OR = 2.29, 95%CI 1.99 – 2.63). Having at least a primary education led to higher odds of being tested for HIV (OR = 1.82, 95%CI 1.53 – 2.15) and having used modern contraception (OR = 1.88, 95%CI 1.60 – 2.21), while having at least a secondary education increased the likelihood of having had the optimal number of antenatal care visits (OR = 1.43, 95%CI 1.18 – 1.87). Regarding the wealth index, the increasing wealth quintiles were positively associated with utilisation of all three SRH services: participants who were richest had higher odds than those who were among the poorest to have had at least four antenatal care visits (OR = 1.81, 95%CI 1.41 – 2.33), tested for HIV (OR = 2.80, 95%CI 2.10 – 3.73) or used modern contraception type (OR = 2.09, 95%CI 1.73 – 2.52).

Religion and region of residence were also significantly associated with SRH service use. Muslims (OR = 0.81, 95%CI 0.70 – 0.94) or the Seven Day Adventist/Pentecostal/Born Again/Evangelical (OR = 0.76, 95%CI 0.66 – 0.86) faith were less likely to have used modern contraception compared to Anglicans, while the Catholic faith did not show any significant association with any of the SRH service use. Women living in West Nile had higher odds of having had at least four antenatal care visits (OR = 1.52, 95%CI 1.05 – 2.21), while they had lower odds of having used modern contraception (OR = 0.50, 95%CI 0.38 – 0.67) compared to people living in the capital. People in East Central were less likely to be tested for HIV (OR = 0.62, 95%CI 0.41 – 0.93) and having used modern contraception (OR = 0.75, 95%CI 0.57 – 0.07). People living in rural areas were less likely to have used modern contraception (OR = 0.86, 95%CI 0.74 – 0.996).

The sensitivity analyses did not reveal any significant differences in the measures of association. The categorization of disability, antenatal care visits, and the use of modern contraception type did not influence the measures of association.

### Effects of interaction terms

In the final models (Table 2), a few interaction terms were statistically significant. Among interactions between disability type and sex, women with difficulty in seeing were less likely to have had HIV testing (OR = 0.56, 95%CI 0.35 – 0.90) compared to men without seeing difficulty, while women with difficulty with self-care had higher odds to have been tested for HIV (OR = 3.68, 95%CI 1.23 – 10.38). Among interactions between disability type and education, only women with hearing difficulty and who had a least secondary education were more likely than women without education to have had at least four and more antenatal care visits (OR = 10.84, 95%CI 1.67 – 70.54). For interactions of disability type and wealth index, women with difficulty seeing in the fourth (OR = 0.15, 95%CI 0.03 – 0.87) and fifth (OR = 0.16, 95%CI 0.03 – 0.89) quintile of wealth index and women with difficulty in communicating in the fourth wealth index quintile (OR = 0.17, 95%CI 0.03 – 0.82) had lower odds of having had at least four antenatal care visits compared to women without any type of disability.

## Discussion

This study found that SRH service use increased among the study population between 2006 and 2016, notably for HIV testing. The study findings further showed the importance of examining the association between disability type – beyond the overall disability status – and SRH service use to discover disparities in SRH service use among people with different impairments. Our results also demonstrate the importance of considering the intersections of vulnerabilities, such as disability, wealth, and sex, in quantitative analyses when examining social determinants of health. Regarding the experience of violence, emotional and physical violence were associated with an increased likelihood of using modern contraception.

Across all three SRH outcomes, the likelihood of service use increased from 2006 to 2016, including for people with disabilities. The years included in our analyses coincided with the 2000-2015 Millennium Development Goals (MDG) which focused on maternal health improvement (MDG 5) including contraception use and the fight against HIV and AIDS (MDG 6) [43]. Among the three outcomes, HIV testing recorded the sharpest increase in 2016 compared to previous years. This can likely be explained by additional HIV financing by The Global Fund in Uganda from 2001 to 2007 [44], and the continuous HIV and AIDS funding by the President’s Emergency Plan for AIDS Relief (PEPFAR) from 2003 to date in sub-Saharan African countries, including Uganda [45]. Furthermore, the 2005 National Policy on HIV Counselling and Testing of Uganda clearly mentions people with disabilities in its roll-out strategies [46]. However, disparities were observed among regions and across the different types of SRH services. Unequal healthcare coverage could potentially have contributed to these regional disparities coupled with slower performance in maternal health outcomes across the country and possibly reflecting a rural-urban divide [47]. A recent study which examined the utilisation of HIV testing and counselling services by women with disabilities during antenatal care in Uganda in 2016 found that women with disabilities, although they accessed the HIV services, were less likely to use them compared to women without disabilities [48].

Our findings showed that social determinants of health, such as education level [17, 18] and wealth [17, 18, 21, 22] were important determinants of SRH service use, with other studies having found that being religious [49, 50] and living in rural areas [17, 20] decreased the likelihood of using some types of SRH services, such as the use of modern contraception. Furthermore, our findings suggest the need to explore beyond individual social determinants of health and consider the multiple layers of coexisting factors. We found that including an interaction between the type of disability, beyond the overall disability status, and other factors such as sex, education level and wealth was important. Considering the different intersectional identities of people enabled us to detect associations and health inequities that would have otherwise been missed. One of these examples of health inequities was the lesser likelihood of richer women with hearing and communication difficulties to have had at least four antenatal care visits relative to poorer women without disabilities. These findings emphasise that people with disabilities are not a monolithic group and are much more that their disability status and can experience intersectional vulnerabilities [51]. Other quantitative research on intersectionality highlighted the “danger of misunderstanding the nature of social experiences and identities manifested in specific contexts” [41] and the importance of adopting the “intersectionality [framework]‘s core ideas of social inequality, power, relationality, social context, and complexity” into quantitative population health research drawing from the social sciences [52]. Based on our qualitative study, we learned from women and men with disabilities that they experienced multiple barriers and layers of discrimination in accessing and using SRH services in Northern Uganda [11].

Specifically, to better understand marginalised people’s realities, the literature on intersectionality has further stressed the need to consider multiple level analysis, from the individual to the populational level [53]. In one of her seminal papers, “When Black + Lesbian + Woman ≠ Black Lesbian Woman” [54], Bowleg recommended examining the non-additive aspects of social identities and power dynamics such as racism, heterosexism, and sexism [53]. According to the context, vulnerable and marginalised people may simultaneously experience privileges on one hand (for example, based on their gender), and disadvantages on another hand (for example, based on race), underlining the importance of looking beyond the additive aspects of social experiences. Our findings suggest that more educated women with hearing difficulties had higher odds than non-disabled women without education to have had optimal antenatal care, while richer women with hearing difficulties had less chances of having the same services. This is possibly due to the combined forces of ableism (a societal system that favours able-bodied people and disadvantages and discriminates against people with disabilities [55, 56]) and other discriminatory power dynamics that could have prevented these women from using the services at the same frequency as other women with other impairments or non-disabled women.

Regarding the experience of violence, although emotional and physical violence were associated with an increased likelihood of using modern contraception, there was no significant interaction between disability and violence. This finding is in contrast to a recent study conducted in Uganda which showed that women with disabilities were significantly more likely to have experienced all forms of violence compared to women without disabilities [57]. Our study may have underestimated the association of the experience of any type of domestic violence on the use of SRH services, due to reasons such as fear of stigma [58] and also because our study population included only ever-married people over the age of 18 years old. A systematic review on gender-based violence victimization in adolescent girls in LMICs reported that young people who were unmarried or married experienced different forms of violence, such as sexual violence, intimate partner violence, and child marriage [59].

### Limitations

Our study has several limitations. The DHS data were collected through self-reporting from participants. However the information related to disability was obtained from the household head for all household members which might have introduced a bias in reporting each household member’s type and level of difficulty in functional dimensions. Moreover, underlying power structures, such as ableism, and the experience of stigma and discrimination were not examined in this study, though they may play an important role in SRH service use and in the experience of SRH services, potentially due to multiple discriminatory barriers hindering the effective use of services among people with disabilities [11]. Intersectional scholarship posits that power systems both structure and reinforce social identities, and could be better understood through mixed methods [53], although qualitative data collection was not a methodological dimension included in the DHS. Finally, structured questionnaires designed for quantitative research, such as the DHS, are not designed to capture diverse societal interactions in various groups, such as people located at the margin of the society [60].

## Conclusion and implications for practice and research

This study provided evidence that SRH outcomes improved over the decade after the approval of the Disability Act in Uganda, including for people with disabilities. Our findings highlight the importance of examining the social determinants of health when studying SRH and the different types of disability. These findings imply that global public health practitioners and researchers cannot consider people with disabilities as a monolithic group. They need to recognize and acknowledge the multiple and coexisting intersecting vulnerabilities people with different types of impairments are experiencing in understanding and analysing data, and in devising SRH services that are inclusive of people with different social identities. Social determinants of health, including disability, are pivotal to the attainment of the SDGs, notably SDG 3 which emphasises universal health coverage, including SRH services, for all people irrespective of their social identities. According to the United Nations’ Convention on the Rights of Persons with Disabilities, disability results from the interactions between people with impairments (physical, sensory, intellectual, and mental) and barriers (physical, attitudinal and structural) in society that hinder their social participation [4]. Provided that accessible environments and/or enabling social determinants of health are present and that barriers are removed [3], people can fully exercise their rights and enjoy more positive health outcomes.

## Data Availability

The datasets used in this study are available online from the DHS Program at: https://dhsprogram.com/data/Using-Datasets-for-Analysis.cfm

## Abbreviations

AIC: Akaike Information Criterion
AIDS: Acquired Immunodeficiency Syndrome
DHS: Demographic and Health Survey
HIV: Human Immunodeficiency Virus
LMIC: Low- and Middle-Income Country
MDG: Millennium Development Goals
PEPFAR: President’s Emergency Plan for AIDS Relief
SDG: Sustainable Development Goals
SRH: Sexual and Reproductive Health
UHC: Universal Health Coverage
UN: United Nations
VIF: Variance Inflation Factor
